# Quercetin as a multifaceted neuroprotective agent against cerebral ischaemia-reperfusion injury: mechanisms and therapeutic potential

**DOI:** 10.3389/fphar.2026.1860351

**Published:** 2026-06-11

**Authors:** Yinyan Wang, Haoran Sheng, Yan Zhang, Xu Guo, Enqi Wu, Yan Chen

**Affiliations:** Neurosurgery, The Second Hospital of Jilin University, Changchun, China

**Keywords:** blood-brain barrier (BBB), cerebral ischaemia-reperfusion injury (CIRI), immunomodulation, neuroprotection, quercetin

## Abstract

Cerebral ischaemia-reperfusion injury (CIRI) is a significant contributor to neurological dysfunction following ischemic stroke, involving multiple pathological mechanisms such as immune disorder, oxidative stress, inflammatory response, and apoptosis. Quercetin, a flavonoid compound widely present in fruits, vegetables, and grains, exhibits multiple biological activities including antioxidant, anti-inflammatory, antiviral, and anti-tumor properties, and has demonstrated significant neuroprotective effects in CIRI models. Currently, systematic reviews on quercetin’s antagonistic effects against CIRI are scarce, and its precise mechanism of action and clinical translational potential remain to be further explored. This article systematically reviews the multidimensional protective mechanisms of quercetin against CIRI, focusing on its action pathways in four dimensions: immune regulation, cell protection, organelle homeostasis maintenance, and blood-brain barrier (BBB) protection. Additionally, it discusses the clinical application prospects, safety, and existing challenges of quercetin, aiming to provide a theoretical basis for subsequent research on quercetin as a neuroprotective strategy and promote its clinical translation in the prevention and treatment of CIRI.

## 1 Introduction

**FIGURE 1:**
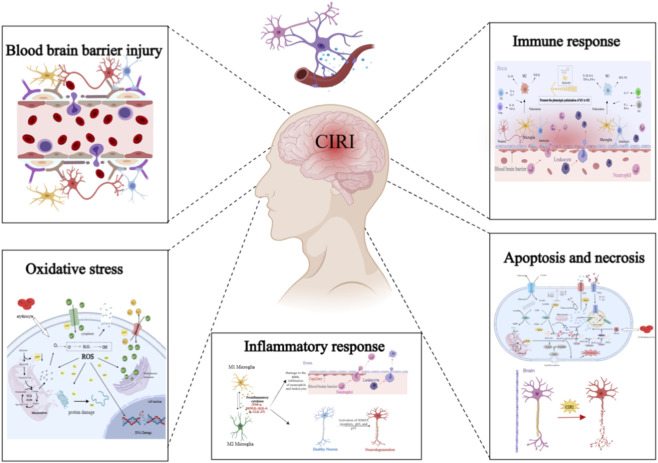
Multiple Pathological Mechanisms of Cerebral Ischemia-Reperfusion Injury (CIRI). The central illustration highlights the CIRI phenomenon within the brain. Surrounding it are depicted five interrelated pathological processes: blood-brain barrier disruption, immune response, oxidative stress, inflammatory response, and apoptosis versus necrosis. Collectively, these contribute to the pathogenesis of CIRI. Created with MedPeer (medpeer.co.uk).

Cerebral ischemic stroke comprises two distinct and consecutive phases, cerebral ischemia(CI) and cerebral ischemia-reperfusion injury(CIRI) (Li X. et al., 2022). These two phases exhibit distinct cellular and molecular mechanisms. During the ischemic phase, acute hypoperfusion leads to rapid ATP depletion, mitochondrial dysfunction, intracellular calcium overload, and acute oxidative stress (Lu et al., 2026). In contrast, cerebral ischemia-reperfusion injury is a significant cause of neurological dysfunction after ischemic stroke (Jo-Watanabe et al., 2024), also involving multiple mechanisms such as immune response, oxidative stress, inflammatory response, and apoptosis (Figure 1) (Chen X. et al., 2025). Immune regulation has become the research hotspot due to therelevant studies for the CIRI pathogenesis in recent years (Li et al., 2025). Research has confirmed that following cerebral ischaemia-reperfusion injury, microglia undergo intense activation and undergo M1/M2 phenotypic polarisation, accompanied by significant activation of the NLRP3 inflammasome (Xu L. et al., 2025). Large amount of damage molecules, including pro-inflammatorycytokines and reactive oxygen species(ROS), have been secreted due tothis injury, which may directly damage neurons and cause BBB degradation (Kunze et al., 2023), enhancing the process of peripheral immune cell penetrating BBB, aggravating braintissue injury, and forming vicious circle.

**FIGURE 2:**
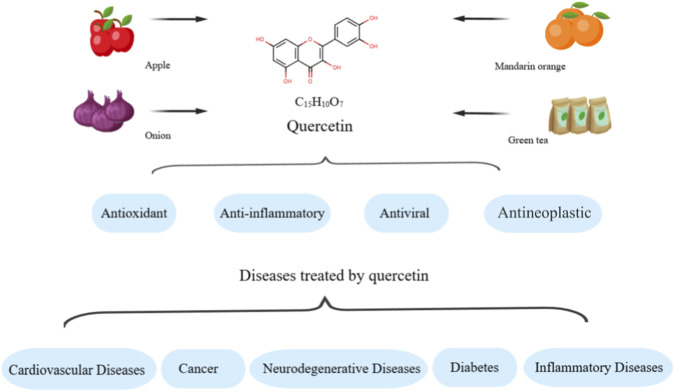
Sources, chemical structure, biological activities, and therapeutic applications of quercetin.Quercetin, a flavonoid with the chemical formula C_15_H_10_O_7_, is naturally derived from foods like apples, onions, mandarin oranges, and green tea. It possesses prominent biological activities including antioxidant, anti-inflammatory, and antiviral effects. These multifaceted activities underpin its potential therapeutic roles in managing cardiovascular diseases, cancer, neurodegenerative diseases, diabetes, and inflammatory diseases. Created with MedPeer (medpeer.co.uk).

Quercetin, a type of flavonoid, can be found ubiquitously in various fruits, vegetables, and grains with high antioxidative, anti-inflammatory, antiviral, and antitumoral capacity (Liu et al., 2022). A large number of studies indicate that quercetin possesses excellent curative efficacy on some chronic diseases such as cardiovascular disease, cancer, neurodegenerative disorders, diabetes, inflammatory states, etc., due to its multifaceted biochemical propertys (Figure 2), for example, quercetin exerts its action as an antioxidative agent through activation of Nrf2 (Sorrenti et al., 2023). In addition to having good antioxidative activity, quercetin can regulate immune responses by using multiple mechanisms (Wu Z. Y. et al., 2025). Quercetin plays a very strong neuroprotective role in the model of CIRI. It regulates microglia polarization, inhibits inflammasome activation, and regulates cytokine network (Mei et al., 2024). Multiple studies indicate that quercetin exhibits potent protective effects against cerebral ischemia-reperfusion injury, providing a reference basis for subsequent in-depth mechanism research and clinical translation (Table 1).

**TABLE 1:** Protective effect and mechanism of Quercetin against cerebral I/R injury.

Study object	Forms of quercetin	Dosage and administration time of quercetin	Model construction	Mechanism	Effect	References
Rats	Intraperitoneal injection	12.5, 25 and 50 mg/kg/day, administered continuously for 3 days	Perfusion 90 min after middle cerebral artery occlusion (MCAO)	Exerts its effects by promoting M2 polarisation of microglia/macrophages through modulation of the PI3K/Akt/NF-κB signalling pathway	Reduced infarct size, improved neurological function	Li et al. (2023)
Rats	Oral gavage	50 mg/kg/day Continuous administration for 4 weeks	Perfusion 30 min after middle cerebral artery occlusion (MCAO)	By activating SIRT1 to reduce endoplasmic reticulum stress, cerebral ischaemia and reperfusion injury is mitigated in hyperglycaemic animals	Neuroprotective effects, reducing neuronal apoptosis	Yang et al. (2025d)
Rats	Oral gavage	10, 30 and 50 mg/kg/day, administered continuously for 5 days	Establishing a GCI/R injury model using the 4-VO method	Downregulation of TLR4 and TRIF protein expression, resulting in diminished release of IL-1β and TNF-α	Effectively alleviated neuronal damage and cerebral oedema	Wang N. et al. (2024)
Rats	Quercetin (oral gavage *in vivo*; medium administration *in vitro*)	*In vivo*: 30, 50 mg/kg/day, continuous administration for 5 days; *In vitro*: 15, 30 μM, treated 2 h before OGD	*In vivo*: Perfusion 30 min after MCAO; *In vitro*: Oxygen glucose deprivation/reoxygenation (OGD/R) model	Activates Nrf2 pathway to enhance antioxidant capacity, reducing OGD/R-induced neurotoxicity and cerebral I/R injury	Attenuated OGD/R-mediated neuronal death, reduced cerebral infarct size, improved neurological function	Wang Y. Y. et al. (2020)
Rats	Glycosylated quercetin (intraperitoneal injection)	20, 40 mg/kg/day, administered continuously for 4 days	Perfusion 90 min after middle cerebral artery occlusion (MCAO)	Enhances solubility and bioavailability via glycosylation, inhibiting neuronal apoptosis and inflammatory response	Enhanced neuroprotective effect vs. unglycosylated quercetin, reduced infarct volume, improved neurological function	Wang et al. (2022)
Rats	Surface-modified engineered exosome-loaded quercetin (intravenous injection)	5, 10 mg/kg (quercetin equivalent)/day, administered continuously for 3 days (1 day before to 1 day after MCAO)	Perfusion 60 min after middle cerebral artery occlusion (MCAO)	Targets delivery of quercetin to impaired neurons, increasing local drug concentration to inhibit neuroinflammation and oxidative stress	Increased quercetin accumulation in ischemic brain, reduced cerebral edema and neuronal damage, improved neurological function	Guo et al. (2021)
Aged rats	Triphenyl phosphonium coated nano-quercetin (oral delivery)	15, 30 mg/kg/day, continuous oral administration for 2 weeks	Age-related global moderate cerebral I/R injury model (4-VO method)	Nano-coating enhances oral bioavailability; triphenyl phosphonium targets mitochondria to reduce mitochondrial oxidative damage	Attenuated age-related exacerbation of cerebral I/R injury, reduced hippocampal neuronal loss, improved cognitive function	Ghosh et al. (2017)
Rats	Quercetin (intraperitoneal injection)	12.5, 25 mg/kg/day, administered continuously for 3 days	Ischemic stroke model (MCAO, 90 min occlusion followed by reperfusion)	Mediates activation of SLC6A3 through Nrf2/STAT3 signaling pathway, exerting neuroprotective effect	Enhanced SLC6A3 expression in ischemic brain, reduced neuronal necrosis, improved neurological deficit	Cai et al. (2025)
Neonatal rats	Quercetin (intraperitoneal injection)	5, 10 mg/kg/day, administered continuously for 4 days	Hypoxia-ischemia induced brain injury model (Rice-Vannucci model)	Inhibits TLR4/NF-κB signaling pathway to reduce inflammatory response and neuronal damage	Attenuated brain atrophy, reduced cortical/hippocampal neuronal apoptosis, improved neurobehavioral outcomes	Wu et al. (2019)
Rats	Eucommia ulmoides Oliv. Bark Extracts (contains quercetin; oral gavage)	100, 200 mg/kg (extract)/day, continuous oral administration for 2 weeks	Perfusion 90 min after middle cerebral artery occlusion (MCAO)	Suppresses microglial activation and inflammatory cytokine (TNF-α, IL-1β) release in cerebral gray matter	Reduced microglial accumulation in gray matter, improved neurological function scores, alleviated cerebral tissue damage	Pan et al. (2025)

**FIGURE 3:**
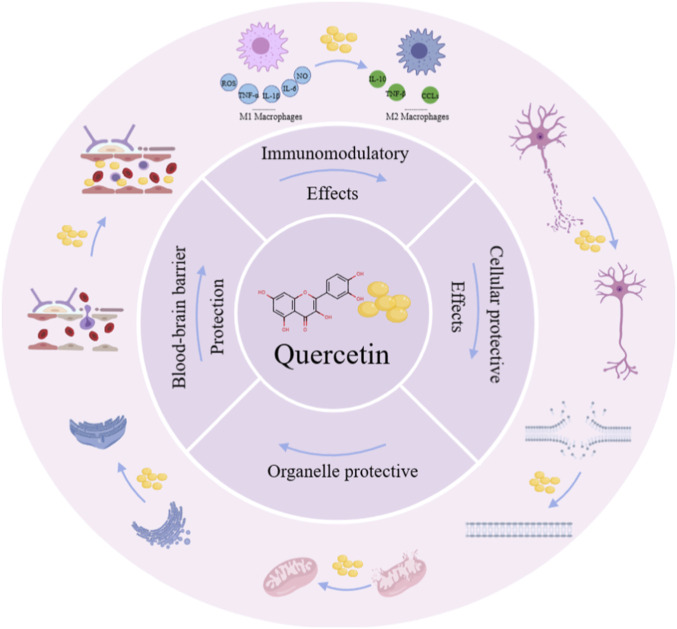
The multidimensional protective mechanisms of quercetin. The core displays the chemical structure of quercetin as a key bioactive flavonoid compound, surrounded by four major protective effects: immunomodulatory effects, cytoprotective effects, organelle protection, and blood-brain barrier protection. These multidimensional actions collectively demonstrate quercetin’s comprehensive protective potential. Created with MedPeer (medpeer.co.uk).

But there are few systematic review studies analyzing the effect of quercetin on CIRI. The exact mechanism remains unclear, and its potential for clinical application requires further exploration. This review comprehensively analyses the multifaceted protective mechanisms of quercetin against CIRI, focusing on its actions in immunomodulation, cellular protection, organelle preservation, and BBB defence (Figure 3). Additionally, it explores the clinical utility of quercetin, aiming to provide a theoretical foundation for future research on quercetin as a neuroprotective strategy and promote its application in CIRI studies.

## 2 Mechanisms of quercetin in counteracting cerebral ischaemia-reperfusion injury

### 2.1 Immunomodulatory effects

#### 2.1.1 Regulation of innate immune responses

The innate immune response acts as the main frontline to protect us from pathogen invasions, which works via a quick, nontargeted means. The chronic inflammatory brain injury (CIRI) will over-stimulate the innate immune system and induce neuronaldamage and the impairment of cognitive function. Quercetin modulates the CIRI-induced innate immune response by multi-mechanisms such as regulatingmicroglia polarization, inhibiting NLRP3 inflammasome activation and regulating complement system, and then relieving neuroinflammation to protect brain tissues (Sun et al., 2025).

**FIGURE 4:**
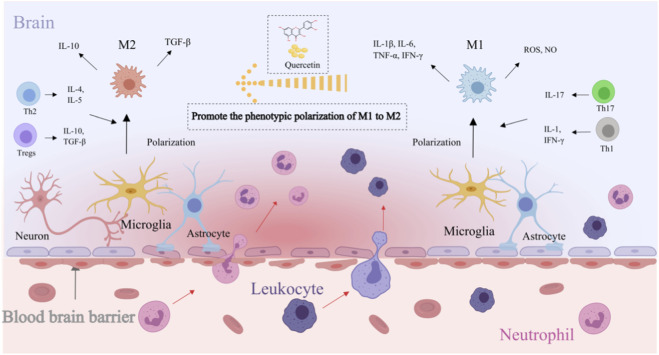
Regulatory Mechanisms of Immune Cell Polarisation in the Central Nervous System Immune Response. This diagram illustrates the process of immune cell polarisation within the central nervous system (CNS) and its regulation by helper T cell (Th cell) subsets. It elucidates the intricate interactions between immune cells, neurons, and the blood-brain barrier through cell polarisation and cytokine signalling pathways in shaping the immune response within the central nervous system. Created with MedPeer (medpeer.co.uk).

Microglia constitute the primary immune cells of the central nervous system (CNS), participating in the function of immune surveillance (Zhou et al., 2025). In CIRI, microglia will react immediately and quickly differentiate into the M1 or M2 state according to various pathological conditions inwhich they are exposed (Figure 4) (Yang Y. et al., 2023). The M1 type microglia have proinflammatory featuresand their activation would be helpful to remove pathogens and clear away dead cells (Peng et al., 2024). However, the persistent overactivity of M1 type microglia can lead tosecondary damage (Peng et al., 2020). M1-type microglia express characteristic molecular markers like CD68 and CD86 and release pro-inflammatory cytokines including TNF-α and IL-1β (Müller et al., 2025), thereby enhancing thelocal inflammatory response that recruits more immune cells and plays an indispensable part during early CIRI (Ye et al., 2025). However, excessive pro-inflammatory activity may cause further neuronal damage, triggering a pathological cytokine storm (Yan et al., 2024). M2 type microglia express CD163 and CD206 (Hoeferlin et al., 2025). Following CIRI onset, they mitigate inflammation and promote tissue repair by secreting anti-inflammatory factors including IL-10 and transforming growth factor-β (TGF-β) (Yang D. et al., 2025). The function of these cells is closely linked to alterations in the brain microenvironment, and is crucial for neuronal protection. Quercetin, inturn, also has an effect in regulating these cells’balance, simultaneously counteracting the innate immunity response initiated by CIRI and alleviating brain tissue damage (Zheng et al., 2025).

**FIGURE 5:**
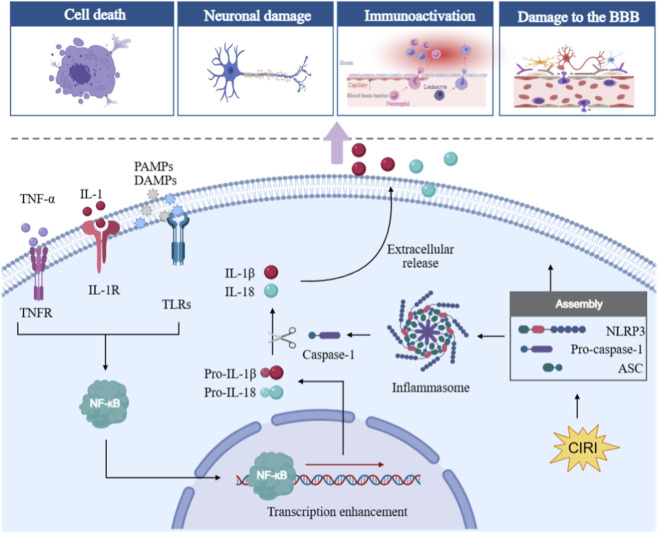
Mechanism of NLRP3 inflammasome activation and its pathological role in cerebral ischaemia-reperfusion injury (CIRI). This diagram elucidates the molecular mechanisms underlying CIRI-induced NLRP3 inflammasome activation and its downstream pathological effects. It depicts how CIRI-induced NLRP3 inflammasome activation orchestrates a cascade of inflammatory and destructive processes within the brain. Created with MedPeer (medpeer.co.uk).

The NLRP3 inflammasome is an important sensor for innate immunity which can control innate immune response regulation, while being activated during CIRI is considered to be one of the most effective inducers of pro-inflammatory reactions (Shaik et al., 2023). By inducing caspase-1, NLRP3 inflammasome can cause the release ofpro-inflammatory cytokines to aggravate neuroinflammation and induce neuronal injury (Figure 5) (Wang et al., 2023a). Quercetin could inhibit the activation of NLRP3 inflammasome and thus prevent the release of IL-1β and IL-18 to achieve ananti-inflammatory effect in post-ischaemia-reperfusion inflammatory injury (Huang et al., 2024). Inhibition of NLRP3 inflammasome can alleviate this problem via directly affecting theprocess of NLRP3 formation or indirectly regulating the signaling pathway, such as the NF-κB signaling pathway (Anjum et al., 2025; Li Q. et al., 2024).

The complement system constitutes a component of the innate immune response, primarily functioning to promote inflammatory reactions, regulate immune responses, and eliminate pathogens (Li and Wu, 2021). During cerebral ischaemia-reperfusion injury (CIRI), complement system activation exacerbates cerebral inflammatory responses, leading to cellular damage and death (Yang H. et al., 2025). Consequently, modulating complement system activity may offer novel therapeutic strategies for the prevention and treatment of CIRI. Complement activation occurs via three pathways, the classical pathway, the alternative pathway, and the mannose-binding lectin (MBL) pathway. Ultimately, all pathways lead to the formation of the membrane attack complex (MAC) to lyse target cells (Laumont et al., 2022). Complement activation simultaneously promotes leukocyte infiltration and the release of pro-inflammatory cytokines, thereby intensifying cerebral inflammatory responses and cellular apoptosis (Yang H. et al., 2024). Quercetin modulates immune responses during CIRI by inhibiting the activation of the classical pathway, alternative pathway, and MBL pathway, thereby reducing the expression of complement proteins C3 and C5.

#### 2.1.2 Modulation of adaptive immunity

In the course of CIRI, adaptive immune responses help regulate the immunological responses to their magnitude and character based on the activation and differentiation of T and B cells (Ning et al., 2022). Moreover, quercetin helps regulate the level of CIRI by modulating T cell subpopulation balance and B cell function.

T cells play an important role in CIRI as mediators of the adaptive immune response (Newell et al., 2013). T cells differentiate into different subsets with pro-inflammatory Th1 and Th17 cells, whereas anti-inflammatory Treg cells function to control inflammation (Lu D. et al., 2025). During CIRI, excessive activation of pro-inflammatory Th1 andTh17 cells contributes to brain tissue damage and neuroinflammation, while poor function of Treg cells amplifies the immune response in an uncontrolled fashion (Ye et al., 2024). Quercetin modulates the balance of T cell subsets, enhancing Treg cell differentiation and blocking Th1 and Th17 cell activity, resulting in restored immunologic homeostasis and reduction of the inflammatory response that occurs during CIRI (Yu et al., 2016).

B cells participate in adaptive immunity mainly by producing antibodies (Shao et al., 2020). B cells not only can mediate humoral immunity, but also can secrete cytokines to regulate immune responses (Wang et al., 2023b). Quercetin suppresses excessive B cell activation and antibody over-production by regulating B cell function (Russo et al., 2014). Quercetin can decrease B cell secretions of pro-inflammatory cytokines (IL-6, TNF-α) (Fan et al., 2025), and thus quercetin has the potential to attenuate B cell-mediated immunologic injury to brain tissue. In conclusion, quercetin can block both the progression and severity of B-cell-mediated immunological brain injury that is relevant for CIRI pathogenesis, while at the same time maintaining normal function of the immune system. Therefore, quercetin may help mitigate adaptive immune response in CIRI patients.

#### 2.1.3 Regulation of cytokine networks

Cytokines play an essential part in causing immune and inflammatory reactions during CIRI (Li et al., 2025). Quercetin regulates the proportion of pro-inflammatory and anti-inflammatory elements via controlling cytokine secretion and signals, thus reducing the inflammatory reaction and promoting cerebral tissue reconstruction and recovery.

Upon onset of CIRI, over-release of pro-inflammatory factors, for example, TNF-α, IL-1β, IL-6, results in neuroinflammation and damage to neurons (Fan et al., 2020). At the same time, insufficient production of anti-inflammatory factors such as IL-10 and TGF-β cannot curb inflammatory response adequately, so immune-mediated injury is intensified (Xia et al., 2023). Quercetin inhibits the expression of pro-inflammatory factors and promotes the secretion of anti-inflammatory factors to mitigate the impact of brain injury and reduce the immune response induced by CIRI and thus protects the nervous system (Fan et al., 2025). Quercetin also regulates cytokine signaling pathways to relieve inflammation in the brain tissue (Wen et al., 2025). For example, it blocks crucial inflammatory signaling pathways such as NF-κB and MAPK and decreases the amount of pro-inflammatory cytokines (Wang Q. et al., 2024).

Chemokines regulate the migration and localization of immune cells during the immune response (Lee et al., 2022). When CIRI happens, the release of large amounts of chemokines causes increased immune cell infiltration and a heightened inflammatory state, whereas quercetin can inhibit this abnormal immune cell infiltration and alleviate local inflammation through chemokine secretion modulation (Yuan et al., 2023). In particular, the expression of chemokines such as MCP-1 is reduced when quercetin regulates chemokine secretion, leading to a reduction in microglia and monocyte infiltration and neuron damage by these infiltrating cells (Wofford et al., 2019). Therefore, quercetin regulates the immune response while decreasing the damage from a hyperactive immune response to prevent the further progression of neuronal injury and promoting brain tissue regeneration (Hou et al., 2025). Quercetin could also regulate interactions between chemokines and their receptors, thus also regulating the movement of immune cells.

### 2.2 Cellular protective effects

#### 2.2.1 Antioxidant stress

**FIGURE 6:**
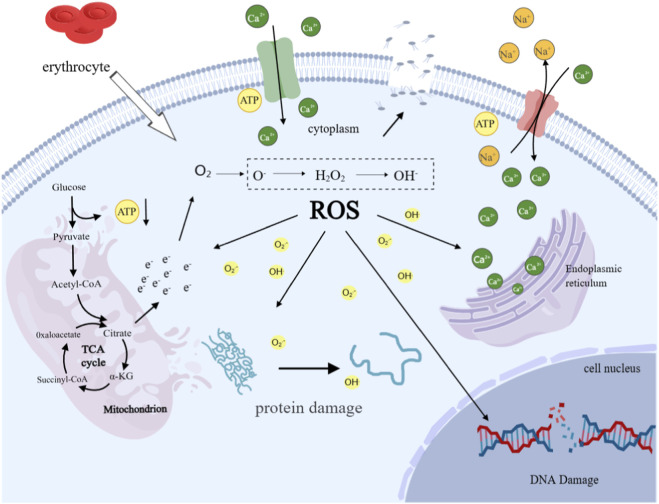
Mechanisms of reactive oxygen species (ROS) generation and their induced cellular damage. This diagram elucidates the pathways of ROS production, comprehensively illustrating how ROS coordinate the initiation of cellular damage cascade reactions through oxidative stress, calcium dysregulation, and macromolecular damage. Created with MedPeer (medpeer.co.uk).

The central nervous system, serving as the command centre of the human body, exhibits high metabolic activity and a relatively elevated basal metabolic rate (Selvaraj et al., 2025). This characteristic necessitates that neurons continuously consume substantial quantities of oxygen and energy substrates under normal physiological conditions to sustain fundamental functions such as nerve impulse conduction and synaptic plasticity (Mohan and Kumar, 2025). In stark contrast to this high metabolic demand, the antioxidant defence system within brain tissue is relatively underdeveloped. The baseline expression levels of key antioxidant enzymes, such as superoxide dismutase (SOD) and glutathione peroxidase (GSH-Px), are markedly lower than in other organs such as the liver and kidneys (Kinoshita et al., 2022). This imbalance between high metabolic expenditure and low antioxidant capacity renders brain tissue more susceptible to oxidative damage during ischaemic and hypoxic events (Jones et al., 2021). When cerebral blood perfusion is abruptly reduced, aerobic metabolic pathways in neuronal cells are rapidly impeded, leading to a sharp decline in adenosine triphosphate (ATP) production and disruption of intracellular energy metabolism (Haupt et al., 2023). Concurrently, hypoxia induces uncoupling of electron transport within the mitochondrial respiratory chain, resulting in the abnormal reduction of oxygen molecules into reactive oxygen species (ROS) (Yin et al., 2025). Owing to the limited scavenging capacity of antioxidant enzymes, excessive ROS rapidly attack phospholipid molecules in cell membranes, triggering lipid peroxidation and disrupting protein structure and function (Battogtokh et al., 2018). Such damage may even directly compromise DNA molecules, leading to irreversible neuronal injury (Figure 6) (Liao et al., 2024). Moreover, this oxidative damage exhibits a cascading amplification effect. The initial ROS surge triggered by the ischaemic event not only directly damages local brain tissue but also exacerbates oxidative stress through secondary pathological mechanisms such as inflammation and blood-brain barrier disruption, thereby perpetuating a vicious cycle (Davinelli et al., 2025).

Quercetin, as a natural antioxidant, derives its efficacy from its unique molecular structure (Henderson et al., 2024). Its conjugated system, comprising three phenolic hydroxyl groups and one catechol group, endows it with potent free radical scavenging capacity (Tsao et al., 2022). The phenolic hydroxyl groups of quercetin act as hydrogen donors, reacting with ROS via electron transfer to convert them into stable non-radical products (Jesus et al., 2023). Furthermore, in peroxidase reactions, transition metal ions (such as Fe^2+^ and Cu^2+^) catalyse ROS generation (Piccirillo et al., 2022). Quercetin’s catechol group forms stable complexes with these ions, thereby blocking the lipid peroxidation chain reaction (Apontes et al., 2014). Quercetin further activates endogenous antioxidant defences by stimulating the Nrf2/ARE signalling pathway, thereby upregulating the activity and gene expression of antioxidant enzymes such as SOD and GSH-Px (Liu L. et al., 2025). Moreover, quercetin enhances energy metabolism and antioxidant capacity following cerebral ischaemia by modulating the expression of the glucose transporter GLUT1 and activating the Sirtuin 1 (SIRT1) pathway (Li et al., 2019).

#### 2.2.2 Regulation of cell death

Apoptosis is a form of programmed cell death regulated by multiple mechanisms, including the mitochondrial pathway and the death receptor pathway (Li X. Q. et al., 2024). Mitochondrial health is closely linked to apoptosis (Tian et al., 2024). Quercetin maintains mitochondrial function and inhibits the release of pro-apoptotic proteins, thereby reducing cell death (Khodakarami et al., 2022). Bax (a pro-apoptotic protein) and Bcl-2 (an anti-apoptotic protein) are key regulators in the mitochondrial pathway (Loke et al., 2023). Quercetin modulates the Bcl-2/Bax ratio by inhibiting the mitochondrial-dependent apoptotic pathway through downregulating Bcl-2 expression and upregulating Bax expression (Erejuwa et al., 2014). Furthermore, quercetin blocks the apoptotic pathway and protects neurons by inhibiting the activation of Caspase-3, Caspase-8, and Caspase-9 (Phoraksa et al., 2023). The PI3K/Akt signalling pathway is crucial for regulating cell survival, proliferation, and apoptosis (Tang et al., 2022). Quercetin enhances cell survival and inhibits apoptosis by activating this pathway (Pang et al., 2025). Activated Akt suppresses pro-apoptotic protein expression while promoting anti-apoptotic protein production, thereby reducing apoptotic levels (Luo et al., 2025). As a key apoptotic regulator, p53 plays a central role in cellular stress responses and DNA damage repair (Dong et al., 2010). Quercetin protects neuronal cells from CIRI-induced death by inhibiting p53 activation and attenuating p53-mediated apoptotic signalling (Kerimi and Williamson, 2018). The JNK and p38 MAPK pathways are closely associated with apoptosis and inflammatory responses (Wree et al., 2018). Quercetin exerts its cytoprotective effects by inhibiting the activation of these pathways and mitigating their pro-apoptotic effects (O'Reilly et al., 2024).

Pyroptosis is a form of programmed cell death initiated by inflammasome activation, typically resulting in cell membrane disruption and the release of numerous cytokines (Zhang et al., 2021). This process is commonly observed in neurodegenerative diseases and CIRI. The NLRP3 inflammasome is the key signal pathway related to pyroptosis (Hu et al., 2025). Quercetin reduced pyroptosis by inhibition of NLRP3 inflammasome formation and activation (Luo et al., 2022). Quercetin can also block the initial step of NLRP3 inflammasome activation to decrease the level of Caspase-1 activation, reducing the release of pro-inflammatory cytokines including IL-1β and IL-18 (Wu B. et al., 2025). Besides, it inhibits Caspase-1 activation to reduce the cleavage of GSDMD in order to alleviate pyroptosis (Chen Y. et al., 2022), as cleavage of GSDMD releases pores to cause membrane rupture (Xiao et al., 2025). Quercetin mitigates pyroptosis by inhibiting this process. The NF-κB pathway serves as a pivotal regulator of inflammatory responses (Chen J. et al., 2022). Quercetin mitigates inflammation by inhibiting NF-κB activation (Lu Y. et al., 2025). Research indicates it reduces NF-κB translocation to the cell nucleus, thereby lowering NLRP3 inflammasome activation levels and subsequently suppressing pyroptosis occurrence (Guan X. et al., 2025).

**FIGURE 7:**
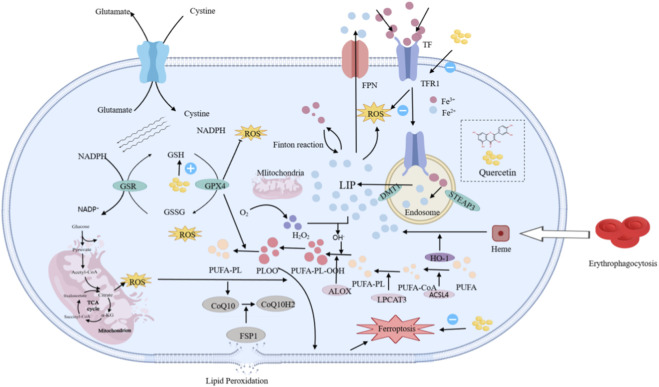
The molecular mechanisms of ferroptosis and its regulation by quercetin. This diagram elucidates the cascading reactions of ferroptosis, encompassing iron metabolism, oxidative stress, lipid peroxidation, and the regulatory role of quercetin. It comprehensively explains the molecular mechanisms of ferroptosis and the protective mechanism by which quercetin counteracts ferroptotic cell death. Created with MedPeer (medpeer.co.uk).

Ferroptosis is an iron-dependent form of cell death characterised by abnormal intracellular iron accumulation and increased lipid peroxidation (Huang et al., 2025). Quercetin effectively counters ferroptosis through multiple mechanisms, including inhibiting lipid peroxide production by modulating antioxidant enzyme activity, thereby reducing lipid peroxidation (Arab et al., 2022). Quercetin further enhances cellular antioxidant defences by elevating glutathione (GSH) levels, thereby slowing the progression of lipid peroxidation (Sharifi-Rad et al., 2023). Concurrently, it reduces intracellular iron accumulation by inhibiting iron uptake and promoting iron storage (Gorini and Vassalle, 2022). This is achieved through downregulating the expression of the transferrin receptor TfR1 and enhancing the function of the iron storage protein ferritin (Figure 7) (Chen S. et al., 2025). Quercetin’s activation of the Nrf2 transcription factor leads to an increase in antioxidant gene expression via the antioxidant response element ARE and an attenuation of ferroptosis-induced cellularinjury (Saidi et al., 2022). Furthermore, quercetin inhibits FASN activity to block excessive fatty acid accumulation and the resultant drop in lipid peroxidation levels (Zhao et al., 2024). Finally, quercetin regulates intracellular calcium levels to prevent the overabundance of calcium. In summary, quercetin affects ferroptosis directly and indirectly, along with affecting other pro-apoptotic and pro-autophagic signaling pathways (Chung et al., 2022).

#### 2.2.3 Regulation of autophagy

Autophagy is a pivotal process for maintaining cellular homeostasis, responding to external stress by degrading and recycling damaged or surplus intracellular components (Wang T. et al., 2024). During CIRI, autophagy plays a dual role: it protects cells by clearing damaged components, yet may induce cell death when overactivated (Wang C. et al., 2020). AMP-activated protein kinase (AMPK), a pivotal energy-sensing enzyme, activates autophagy during energy deprivation (Hu et al., 2021). Quercetin promotes autophagy by activating AMPK and inhibiting the mTOR signalling pathway (Guan Y. et al., 2025). As a negative regulator of autophagy, mTOR is inhibited by quercetin, thereby releasing autophagy suppression and promoting its initiation (Raghav and Jeong, 2024). This assists cells in clearing damaged components and alleviating CIRI (Liu et al., 2025b).

Beclin-1 plays a pivotal role in autophagosome formation, whilst LC3 conversion serves as a key autophagic marker (Wu et al., 2023). Quercetin enhances autophagy by increasing Beclin-1 expression and promoting LC3 conversion, thereby protecting cells from injury (Wong et al., 2023). The PI3K/Akt pathway typically maintains cell survival by inhibiting autophagy (Xu J. et al., 2025). Quercetin enhances autophagy by suppressing PI3K/Akt pathway activation, thereby counteracting its inhibitory effect on autophagy (Yang S. et al., 2024). This mechanism enables quercetin to enhance cellular stress responses and promote clearance of damaged components under ischaemia-reperfusion stress. P53, a pivotal factor in cellular responses to oxidative stress and DNA damage, promotes autophagy by regulating autophagy-related gene expression (Debnath et al., 2023). Quercetin further modulates autophagy by enhancing p53 activity (Yang Y. H. et al., 2023). Studies indicate it promotes p53-mediated autophagy, aiding cellular clearance of damaged components during CIRI. The ATG protein family (e.g., ATG5, ATG7, ATG12) plays a pivotal role in autophagy (Jin et al., 2024). Quercetin enhances autophagy by upregulating ATG family protein expression (Janda et al., 2021), thereby promoting autophagosome formation and the clearance of intracellular debris or damaged components (Li et al., 2021), thus facilitating cellular functional recovery following CIRI.

The balance between autophagy and apoptosis is important in CIRI (Li et al., 2020). Quercetin maintains autophagy’s physiological function by regulating its interaction with apoptosis (Qiang et al., 2022), thereby preventing cellular damage arising from either excessive autophagy or its inhibition. Specifically, while enhancing autophagy, quercetin reduces apoptotic signals such as Caspase-3 activation, thereby diminishing apoptosis and achieving a cell-protective effect (Carecho et al., 2023). Furthermore, quercetin modulates autophagy by regulating specific microRNAs (miRNAs) (Toma et al., 2020), such as upregulating autophagy-associated miRNAs, thereby promoting autophagy and enhancing cellular tolerance to CIRI.

### 2.3 Organelle protection

#### 2.3.1 Mitochondrial protection

Mitochondrial dysfunction is a primary mechanism triggering cellular injury and death during CIRI (Zhong et al., 2025). Quercetin protects mitochondrial function and integrity through multiple pathways (Jurcau and Jurcau, 2023), thereby reducing mitochondria-related cell death (Fakhri et al., 2022).

Loss of mitochondrial membrane potential constitutes a critical hallmark of apoptosis (Li Y. et al., 2022). Quercetin inhibits the decline in mitochondrial membrane potential, thereby maintaining mitochondrial stability and preventing the release of pro-apoptotic molecules such as cytochrome C (Zalpoor et al., 2022). This action curtails the activation of mitochondria-dependent apoptotic pathways (Cimmino et al., 2023). Mitochondria serve as the primary intracellular source of ROS (Contreras et al., 2025); excessive ROS production induces oxidative stress, damaging mitochondrial membranes and proteins (Yang et al., 2025c). Quercetin maintains the oxidative stress levels by increasing antioxidant activity, reducing ROS production, and relieving oxidative damage (Zhang L. et al., 2025). It attenuates apoptotic signalling within the mitochondrial pathway by upregulating anti-apoptotic proteins (Bcl-2, Bcl-xl) and inhibiting the activation of pro-apoptotic protein Bax (Xie et al., 2023), thereby preserving mitochondrial membrane integrity and enhancing cellular survival (Xiong et al., 2024). In addition, mitochondrial calcium overload has been recognized as an important event associated with the damage of mitochondria. The decreased release of calcium ions due to quercetin provides relief to cellular stress generated by an excessive calcium load in mitochondria, while protection against the rupture of mitochondria membrane and cell death is possible due to this decrease (Abdel-Rahman et al., 2021).

Caspase-3 and caspase-9 are the major effectors of apoptosis via mitochondrial-dependent apoptosis pathways (Le Clorennec et al., 2023). By blocking the activation of these enzymes, quercetin prevents damage to the mitochondria during apoptosis and thus protects the mitochondrial structural and functional integrity (Zhang et al., 2023). Mitochondrial autophagy plays an important role in mitochondrion function and quality control (Pearson and Soleimanpour, 2018). Quercetin has been shown to promote mitochondrial clearance through its action in modifying the transcriptional level of autophagy-related genes to preserve the number and functionality of healthy mitochondria (Xiong et al., 2024). Excessive opening of the mitochondrial permeability transition pore (mPTP) is closely related to mitochondrial dysfunctions and cell death (Xia et al., 2024). Quercetin can inhibit the opening of mPTP, which will maintain the integrity of the mitochondrial membrane, prevent the release of pro-apoptotic molecules and reduce mitochondrial-mediated cell death (Zhang Q. et al., 2025). Quercetin increases the levels of uncoupling proteins (UCPs) and other mitochondrial-associated proteins that increase the expression of proteins which regulate both the mitochondrial protein synthesis and the repair processes, thereby reinforcing the repair mechanisms of mitochondria (Nunn et al., 2023). Furthermore, it modifies the components of the mitochondrial membrane to ensure stable mitochondrial structures, reduces mitochondrial membrane injury caused by lipid peroxidation and improves membrane stability (Abo-Saif et al., 2025).

Mitochondrial DNA (mtDNA) damage is related to mitochondrial function disturbance (Reddyvari et al., 2017). Quercetin activates mtDNA repair mechanisms, promoting the repair of damaged mtDNA and safeguarding normal mitochondrial function, thereby reducing cell death (Kratz et al., 2021). Furthermore, quercetin regulates mitochondrial energy metabolism, enhancing mitochondrial ATP synthesis capacity (Zhang Q. et al., 2025). This improves post-ischaemic mitochondrial energy deficiency, maintains normal cellular metabolism, and supports cellular recovery and survival (Guan Y. et al., 2025).

#### 2.3.2 Endoplasmic reticulum (ER) protection

Endoplasmic reticulum stress (ERS) is a main mechanism that leads to cell death during the CIRI process (Liu et al., 2025c), and mainly originates from changes within and outside the cells like oxidative stress, calcium balance disturbance, and nutrient starvation which triggers unfolded protein response (UPR). Quercetin regulates ERS via multiple manners, so as to reduce the damage caused by ERS (Lu Y. et al., 2025). The principal response to ERS involves the activation of three major signaling pathways (IRE1, PERK, ATF6) (Katsuyama et al., 2024). Activation of these pathways aids cellular adaptation to increased endoplasmic reticulum load, yet excessive activation leads to cell death (Wang et al., 2025). Quercetin reduces ERS-induced cell damage by decreasing overactivation of these pathways (Zhang et al., 2026). Research suggests that quercetin inhibits IRE1α activity, thereby reducing the nonsense mRNA splicing of X-box-binding protein-1 (XBP1), hence hindering ERS-induced inflammation and apoptosis (Saadh et al., 2025).

PERK is a pivotal kinase in the ERS response. Activated PERK inhibits protein translation by phosphorylating eukaryotic initiation factor 2α (eIF2α), thereby alleviating endoplasmic reticulum burden and initiating anti-apoptotic responses (Perea et al., 2023). Quercetin reduces eIF2α phosphorylation by inhibiting PERK activation, thus mitigating ERS-induced cell death (Zhang et al., 2026). Furthermore, quercetin modulates the expression of activator of transcription factor 4 (ATF4), mitigating its pro-apoptotic effects during endoplasmic reticulum stress and thereby further inhibiting cell death (Fontana et al., 2020). ATF6, a pivotal transcription factor in the endoplasmic reticulum stress response, triggers the expression of downstream stress response genes such as C/EBP homologous protein (CHOP) upon activation (Halilovic et al., 2024). Quercetin alleviates endoplasmic reticulum stress-induced apoptotic signalling by inhibiting ATF6 activation and reducing CHOP expression (Gu et al., 2022).

Oxidative stress is considered the principal instigator of endoplasmic reticulumstress (ERS) (Ren et al., 2023). Moreover, quercetin alleviates ERS by lessening the degree ofoxidative stress (Aldrich et al., 2023). As an intracellular calcium ion reservoir, the endoplasmic reticulum maintains calcium homeostasis through the calcium ATPase (SERCA) pump (Zhang et al., 2020). Quercetin promotes calcium ion influx into the endoplasmic reticulum by inhibiting SERCA function (Cui et al., 2022). This mechanism alleviates ER damage caused by calcium overload and mitigates the ERS response (Peyman et al., 2023). ER autophagy represents a crucial cellular mechanism for clearing damaged or over-expanded ER via the autophagic pathway (Liu et al., 2024). Quercetin promotes ER autophagy by enhancing the expression of ATG family proteins, thereby accelerating the clearance of damaged ER structures and reducing the impact of ERS on cells (Tsui et al., 2024). ERS typically induces cell death by activating pro-apoptotic signals (Li M. et al., 2024). Galactosidase-12 constitutes a pivotal component of the ER stress-induced apoptosis pathway. Quercetin mitigates ER stress-induced apoptosis by inhibiting galactosidase-12 activation.

### 2.4 Blood-brain barrier (BBB) protection

Quercetin, a natural plant flavonoid, exerts multiple protective effects on the BBB during CIRI (Sun et al., 2024). It preserves BBB integrity by inhibiting inflammatory responses and reducing BBB permeability (Nájar et al., 2023). Oxidative stress is a major factor in BBB disruption (Hu et al., 2022). Quercetin protects the BBB by enhancing the activity of antioxidant enzymes and reducing the production of harmful substances (e.g., H_2_O_2_) (Zhang et al., 2024). Moreover, quercetin maintains the structure and function of the blood-brain barrier by regulating the expression and distribution of tight junction proteins such as occludin, tight junction protein-5, and tight junction protein-1. It mitigates neuronal damage induced by CIRI by inhibiting endogenous and exogenous apoptotic pathways (Huang et al., 2024). Reducing the number of apoptotic cells contributes to preserving blood-brain barrier integrity (Ribierre et al., 2024). Furthermore, quercetin regulates vascular endothelial growth factor (VEGF) synthesis and nitric oxide (NO) synthesis for repairing and stabilizing BBB. Matrix metalloproteinases (MMPs) play a pivotal role in CIRI-induced BBB damage (Jin et al., 2019). MMP activation degrades the extracellular matrix of the blood-brain barrier, leading to structural damage (Ungvari et al., 2021). Quercetin can prevent this type of structural damage by inhibiting MMP activity, thereby reducing the degradation of the matrix (Medoro et al., 2025).

## 3 Safety dose and toxicology of quercetin

Quercetin is a naturally occurring flavonoid which has been identified with several potential beneficial uses, due to its wide range of biological actions and lower potential toxicity (Medoro et al., 2025). The evaluation of safety concerning its therapeutic use should include quantification of the adverse side effects from long-term or high dose application (Bukhari, 2022).

The bioavailability of quercetin is relatively low (approximately 1%–2%), primarily attributable to its poor water solubility, low intestinal absorption rate, and first-pass metabolism (Huang et al., 2024). Following oral administration, quercetin undergoes microbial metabolism in the gut to form aglycone quercetin, which is further metabolised via glucuronidation and sulphation into metabolites with reduced biological activity (Zhu et al., 2024; Alattar et al., 2023). To enhance bioavailability, strategies such as nanocarriers (e.g., liposomes, polymeric nanoparticles) and structural modifications are currently under active development (Hashemi and Sadowski, 2020). For example, compared to the original quercetin nanoparticles, liposome encapsulation increased their solubility by a factor of 44 (Parhi et al., 2020). Concurrently, animal studies demonstrate quercetin’s exceptional safety profile. Acute toxicity studies of quercetin indicate that a single intravenous injection of 100–150 mg/kg/day produced no toxicological symptoms (Chen B. et al., 2022). Human studies indicate that no adverse reactions were observed following oral administration of 1g of quercetin daily for three consecutive months (Chu et al., 2020). Although quercetin exhibits a high degree of overall safety, studies indicate that long-term administration (58 and 104 weeks) of extremely high doses (1,000 and 40,000 ppm) may lead to increased carcinogenic activity in rats (Cunningham et al., 2022). Individual metabolic variability and potential risks associated with long-term use, such as interactions with other medications, warrant further attention. Future research should focus on enhancing targeted delivery efficiency and validating long-term safety through carefully designed clinical trials.

## 4 Challenges and future directions

The protective effects of quercetin against CIRI are primarily mediated through multiple mechanisms, including immunomodulation, anti-inflammatory action, antioxidant properties, anti-apoptotic effects, and blood-brain barrier protection. Significant challenges remain in translating quercetin from preclinical research to clinical application. The structure–activity relationship (SAR) between quercetin and isoquercitrin is of great significance for the translational application of neuroprotective flavonoids. Isoquercitrin features a 3-O-glucosylation modification, which not only elevates aqueous solubility but also facilitates active intestinal absorption through the sodium-glucose linked transporter 1 (SGLT1), thereby markedly improving systemic and cerebral bioavailability relative to aglycone quercetin. Recent pharmacokinetic studies have confirmed that, compared to quercetin, oral administration of isoquercetin results in 1.7- to 10-fold higher plasma drug concentrations, along with a shorter time to peak concentration (Tmax) and a larger area under the curve (AUC). In experimental cerebral ischemia-reperfusion injury models, equimolar isoquercitrin more potently suppresses excessive reactive oxygen species generation, inhibits the NF-κB/MAPK inflammatory signaling cascade, and modulates Bcl-2/Bax-dependent apoptotic pathways than quercetin. In terms of its mechanism of action, isoquercetin undergoes rapid deglycosylation in the intestine, releasing free quercetin as the primary bioactive compound. In this regard, isoquercetin, as a prodrug, exhibits higher delivery efficiency. These SAR and metabolomics findings highlight the potential of isoquercetin as an optimized brain-targeted drug for the treatment of CIRI. Similarly, while nanocarriers show significant potential for addressing issues such as low bioavailability and the blood-brain barrier, their clinical feasibility requires further validation. Furthermore, most preclinical studies utilize young, healthy animal models, which may not fully reflect the clinical characteristics of CIRI patients, particularly elderly patients with comorbidities such as hypertension or diabetes. Future research should explore the application prospects of quercetin and isoquercetin, various quercetin nanoparticle formulations, and brain-targeted delivery systems in clinically relevant ischemia-reperfusion injury models. Prospective clinical trials should also be designed to confirm the pharmacokinetic profiles, therapeutic efficacy, and safety characteristics of quercetin in patients with ischemia-reperfusion injury.

In conclusion, quercetin exerts beneficial effects on cerebral ischaemia-reperfusion injury via various pathways. Its ability to modulate immune responses, relieve inflammation, reduce oxidative stress, protect cell structure, and maintain the blood–brain barrier function signifies it as a possible treatment option in CIRI-related therapies. Although results from some *in vitro* and *in vivo* studies provided preliminary evidence for quercetin’s therapeutic benefits against CIRI, taking quercetin from preclinical studies to clinical trials still pose several challenges, such as poor bioavailability, demand for improved drug delivery methods, limited long-term human safety and efficacy data, etc. Furthermore, recruiting a larger number of participants from diverse populations in future clinical trials could provide additional support.

To sum up, quercetin is a candidate to be employed as an element in efficient CIRI preventive and therapeutic regimens, but studies are necessary to deal with limitations as described above before demonstrating clinical relevance in neurological pathologies.
